# Discovery of a New Phase in Thin Flakes of KV_3_Sb_5_ under Pressure

**DOI:** 10.1002/advs.202415012

**Published:** 2025-02-28

**Authors:** Zheyu Wang, Lingfei Wang, King Yau Yip, Ying Kit Tsui, Tsz Fung Poon, Wenyan Wang, Chun Wai Tsang, Shanmin Wang, David Graf, Alexandre Pourret, Gabriel Seyfarth, Georg Knebel, Kwing To Lai, Wing Chi Yu, Wei Zhang, Swee K. Goh

**Affiliations:** ^1^ Department of Physics The Chinese University of Hong Kong Shatin Hong Kong China; ^2^ Department of Physics Southern University of Science and Technology Shenzhen Guangdong China; ^3^ National High Magnetic Field Laboratory Florida State University Tallahassee FL USA; ^4^ Univ. Grenoble Alpes, CEA, Grenoble‐INP, IRIG, Pheliqs Grenoble 38000 France; ^5^ Univ. Grenoble Alpes, INSA Toulouse, Univ. Toulouse Paul Sabatier, EMFL, CNRS, LNCMI Grenoble 38042 France; ^6^ Department of Physics City University of Hong Kong Kowloon Hong Kong China

**Keywords:** kagome superconductors, mobility spectrum analysis, pressure‐induced phase, quantum oscillations

## Abstract

Results of magnetotransport measurements are reported on KV_3_Sb_5_ thin flakes under pressure. The zero‐field electrical resistance reveals an additional anomaly emerging under pressure (*p*), marking a previously unidentified phase boundary T*(*p*). Together with the established *T*_CDW_(*p*) and *T*_*c*_(*p*), denoting the charge‐density‐wave transition and a superconducting transition, respectively, the temperature‐pressure phase diagram of KV_3_Sb_5_ features a rich interplay among multiple phases. The Hall coefficient evolves reasonably smoothly when crossing the T* phase boundary compared with the variation when crossing *T*_CDW_, indicating the preservation of the pristine electronic structure. The mobility spectrum analysis provides further insights into distinguishing different phases. Finally, the high‐pressure quantum oscillation studies up to 31 T combined with the density functional theory calculations further demonstrate that the new phase does not reconstruct the Fermi surface, confirming that the translational symmetry of the pristine metallic state is preserved.

## Graphical abstract

The discovery of an unexpected new phase is reported in thin flake of KV_3_Sb_5_ under pressure. The new phase, alongside the CDW and superconducting phases, reveals an intriguing interplay between various novel phases in the kagome system. These findings suggest that this phase is related to nematicity, showcasing the kagome system's potential for stabilizing novel phases intertwining with superconductivity.

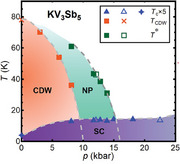

## 1 Introduction

The kagome lattice, a 2D network of corner‐sharing triangles, intrinsically accommodates Dirac fermions, van‐Hove singularities (vHs) and flat bands.^[^ ^1^, ^2^, ^3^, ^4^, ^5^, ^6^ ^]^ Due to the diverging density of states at the vHs and flat bands, a variety of correlated many‐body ground states can be realized. In kagome metal AV_3_Sb_5_ (A = K, Rb, Cs), both charge density wave order^[^ ^7^, ^8^, ^9^, ^10^, ^11^ ^]^ (*T*_CDW_∼80–100 K) and superconductivity^[^ ^3^, ^12^, ^13^ ^]^ (*T*_c_∼0.9–2.7 K) have been revealed by various measurements. The charge density wave (CDW) phase has attracted significant attention for its unconventional characteristics, in particular, whether time‐reversal symmetry breaking (TRSB) occurs within the CDW phase.^[^ ^14^, ^15^, ^16^, ^17^, ^18^, ^19^, ^20^ ^]^ While the issue of TRSB remains under debate, the understanding of the CDW superlattice has gained substantial progress. Regarding in‐plane distortion, both “Star of David” and trihexagonal patterns can be adopted^[^ ^21^ ^]^ and different stacking arrangements along *c*‐direction are observed among KV_3_Sb_5_, CsV_3_Sb_5_, and RbV_3_Sb_5_, indicating the 3D nature of the CDW order.^[^ ^8^, ^22^, ^23^ ^]^ Besides, a stripe order of CDW with a modulation of 4*a*_0_ has been revealed by the detailed scanning tunneling microscopy (STM) measurement^[^ ^24^ ^]^ and a commensurate CDW to incommensurate CDW (ICCDW) transition under pressure has been proposed by the nuclear quadrupole resonance (NQR) measurement.^[^ ^25^ ^]^ The interplay between CDW and superconductivity also presents intriguing dynamics. While the CDW order is suppressed monotonically as pressure increases, the double‐dome feature of *T*_c_ has been discovered across all three compounds,^[^ ^26^, ^27^, ^28^ ^]^ with *T*_c_ reaching a maximum near the CDW suppression point, suggesting the potential role of charge fluctuations on superconductivity.^[^ ^29^ ^]^

Despite the ongoing debate on the nature of the unconventional CDW and superconductivity, more recent studies have expanded the general interest to examine their interplay with other correlated phenomena, notably the possibility of electronic nematicity. While the elastoresistance,^[^ ^30^ ^]^ nuclear magnetic resonance,^[^ ^31^ ^]^ STM^[^ ^32^, ^33^ ^]^ and zero‐field muon spin relaxation measurements^[^ ^34^ ^]^ have pointed toward the presence of the nematic order with *C*_2_ symmetry inside the CDW phase, a recent high‐resolution torque measurement in CsV_3_Sb_5_ unveiled an odd‐parity nematic phase^[^ ^35^ ^]^ above *T*_CDW_. Furthermore, in‐plane transport measurements on CsV_3_Sb_5_ and RbV_3_Sb_5_ have uncovered a twofold symmetry of the superconducting phase,^[^ ^36^, ^37^ ^]^ indicating the intimate interplay between nematicity and superconductivity.

In this manuscript, we report the unexpected emergence of a new phase in KV_3_Sb_5_ under pressure. The anomaly representing the transition temperature manifests in the transport data at low pressure and decreases monotonically as pressure increases. The relatively smooth evolution of the Hall coefficient when cooling across the phase boundary indicates the absence of a severe Fermi surface reconstruction, and the mobility spectrum analysis derived from the magnetotransport data offers a deeper insight to distinguish the new phase from other features. Finally, we present the quantum oscillation and density functional theory calculation results to confirm that the pristine Fermi surface of KV_3_Sb_5_ is preserved in the new phase. Our result suggests that nematicity is the leading candidate of the new phase. The newly constructed temperature‐pressure phase diagram uncovers an intricate interplay among the new phase, CDW and the superconductivity in the kagome system and reaffirms that kagome lattices provide a novel platform to stabilize novel phases that are often intertwined with superconductivity.

## 2 Experimental Results

### 2.1 Emergence of a Pressure‐induced Phase in KV_3_Sb_5_ Thin Flakes

**Figure 1:**
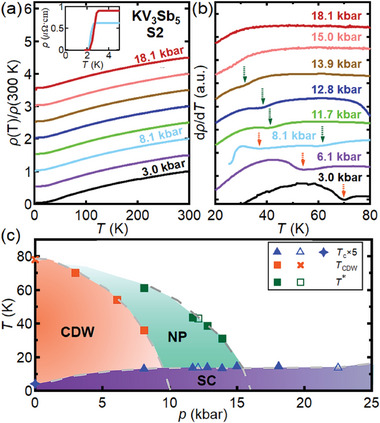
Temperature dependence of (a) the renormalized resistivity ρ(*T*)/ρ(300K) and (b) dρ/d*T* of S2 at different pressures. While the curves here are shifted vertically for clarity, data plotted in the absolute unit can be found in the Supporting Information.^[^ ^38^ ^]^ The inset in (a) displays the superconducting transition at 8.1 and 18.1 kbar, while the orange solid arrows and the green dashed arrows in (b) respectively represent *T*_CDW_ and T*. For dρ/d*T* down to 10 K at 8.1 kbar, see Figure S6c. (c) Temperature‐pressure phase diagram of KV_3_Sb_5_. The solid symbols represent data from S2 and the open symbols are from S1. The star and the cross are ambient‐pressure *T*_c_ and *T*_CDW_ taken from Ref. [39], respectively. The *T*_c_ is multiplied by 5.

**Figure** **1**a shows the temperature dependence of the resistivity collected on S2 at various pressures, while the corresponding dρ/d*T* curves are presented in Figure 1b. At 3.0 kbar and 6.1 kbar, dips in dρ/d*T* are recorded respectively at 70 and 54 K (see orange arrows in Figure 1b). These features can be smoothly connected to the anomaly at 78 K observed in another thin flake reported in our earlier work at ambient pressure,^[^ ^39^ ^]^ where we have established it to be a CDW phase transition. Thus, the anomaly at 3.0 and 6.1 kbar are attributed to CDW phase transition temperatures *T*_CDW_. At 8.1 kbar, however, three clear features can be observed: in addition to the superconducting transition at *T*_c_ = 2.6 K that can be unambiguously identified because of the zero resistance (see inset of Figure 1a), dρ/d*T* displays two minima instead of just one dip at higher temperatures, as indicated by the green and orange arrows in Figure 1b, respectively. The dip at the lower temperature (36 K) extends the decreasing trend of *T*_CDW_ recorded at lower pressures. Therefore, it is natural to assign the anomaly at the lower temperature to *T*_CDW_. The anomaly at the higher temperature (61 K), on the other hand, have not been reported in the literature.

To learn more about the newly‐observed anomaly at 8.1 kbar, we continue to increase the pressure. At 11.7 kbar, a clear minimum in dρ/d*T* is recorded at 43.5 K, between two minima at 8.1 kbar. Since *T*_CDW_ in all three AV_3_Sb_5_ systems have been reported to be a monotonically decreasing function of *p*,^[^ ^28^, ^40^, ^41^ ^]^ it is unreasonable to assign the local minimum at 11.7 kbar to *T*_CDW_, as this would imply a sudden increase of *T*_CDW_ under pressure. Instead, this anomaly must be connected to the one observed at 8.1 kbar. It is now clear that a new temperature scale emerges in KV_3_Sb_5_ under pressure, to which we attach a symbol T*. Further increasing the pressure, a similar anomaly in dρ/d*T* can be followed to 13.9 kbar, enabling the construction of T*(*p*), which smoothly connects four datapoints in S2. At 15 kbar, no obvious dip can be discerned.

Figure 1c shows the temperature‐pressure phase diagram constructed for thin flakes of KV_3_Sb_5_, including various anomalies detected. While *T*_CDW_ diminishes at lower pressures, T* persists to a higher pressure and extrapolates to 0 K at around 17 kbar. We stress that T* is observed in both S1 and S2: in S1, T* is 43 K at 12.1 kbar (open square in Figure 1c), in an excellent agreement with the pressure dependence of T* recorded in S2. Concomitant with the pressure evolution of *T*_CDW_ and T*, *T*_*c*_ with values greater than 2 K can also be observed for pressures greater than 8.1 kbar. Thus, Figure 1c is suggestive of an intriguing interplay among superconductivity, CDW and the new phase (NP) demarcated by T*.

### 2.2 Evolution of the Hall Coefficient under Pressure

**Figure 2:**
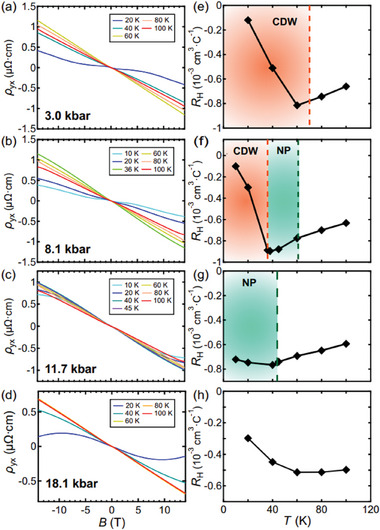
Hall resistivity against the magnetic field at different temperatures for S2 at 3.0 kbar (a), 8.1 kbar (b), 11.7 kbar (c) and 18.1 kbar (d), respectively. Panels (e–h) depict the temperature dependence of the Hall coefficients at corresponding pressures. The orange and the green shaded areas represent the CDW phase and the T*‐related phase respectively.

To investigate the new anomaly, we trace Hall signals of S2 at representative pressures and temperatures, depicted in **Figure** **2**a–d. At 3.0 kbar, where only *T*_CDW_ exists, a characteristic “S”‐shape feature appears in the low‐field region of ρ_yx_ at 20 K, but the feature diminishes above *T*_CDW_. This feature is consistent with the reported anomalous Hall effect (AHE) in AV_3_Sb_5_.^[^ ^14^, ^15^, ^16^, ^42^ ^]^ At 80 K, ρ_yx_ is linear in field up to ±14 T without the “S”‐shape feature, indicating the system is in the pristine phase. Then we extract the Hall coefficient *R*_H_ from the slope of ρ_yx_(*B*) at low field using a one‐band approximation. For data distorted by the “S”‐shape feature, *R*_H_ is calculated from the slope of ρ_yx_(*B*) just beyond the “S”‐shape region, following a procedure introduced in previous works on AV_3_Sb_5_ (see, e.g., Ref. [14]). As shown in Figure 2e, |*R*_H_| shows a maximum when crossing *T*_CDW_, and |*R*_H_| significantly decreases with further cooling. Since the carrier density is inversely proportional to |*R*_H_|, the temperature dependence of |*R*_H_| is consistent with the evolution of the carrier density reported at ambient pressure in Ref. [14].

At 8.1 kbar, at which both T* and *T*_CDW_ are detected in the normal state, the “S”‐shape feature can still be observed at 10 K and 20 K but is greatly weakened. Between *T*_CDW_ (36 K) and T* (61 K), the Hall signals no longer host the “S”‐shape feature. Instead, ρ_yx_ is linear up to 10 T, and only deviates slightly from linearity beyond 10 T. Above T*, ρ_yx_ shows linear dependence on field up to 14 T. As displayed in Figure 2f, |*R*_H_| extracted with the same procedure again shows a maximum near *T*_CDW_. Surprisingly, *R*_H_ changes reasonably smoothly when crossing T* compared with the variation when crossing *T*_CDW_, indicating that T* does not significantly affect the electronic structure.

Next, we increase the pressure to 11.7 kbar where only T* appears in the normal state. Instead of the “S”‐shape feature, ρ_yx_ shows linear behavior up to 5 T and deviates from the linearity at higher fields at 10 K and 20 K. For *T* > 40 K, ρ_yx_ shows perfect linear dependence on the field. We show the extracted *R*_H_ in Figure 2g. Once again, |*R*_H_| does not show a significant decrease across T*. Finally, we investigate S2 at 18.1 kbar where no anomaly appears in the normal state. From the low‐field slope of ρ_yx_(*B*) at various temperatures plotted in Figure 2d, *R*_H_ is extracted and displayed in Figure 2h. At 18.1 kbar, *R*_H_ shows a weak temperature dependence, and it is worth mentioning that the magnitude of changes in *R*_H_ in Figure 2h is comparable to the changes in *R*_H_ at 11.7 kbar at which T* exists. Taking into account all these data, we conclude that the new feature associated with T* does not appear to modify the electronic structure.

### 2.3 Mobility Spectrum as a Diagnostic Tool for Phase Transitions

**Figure 3:**
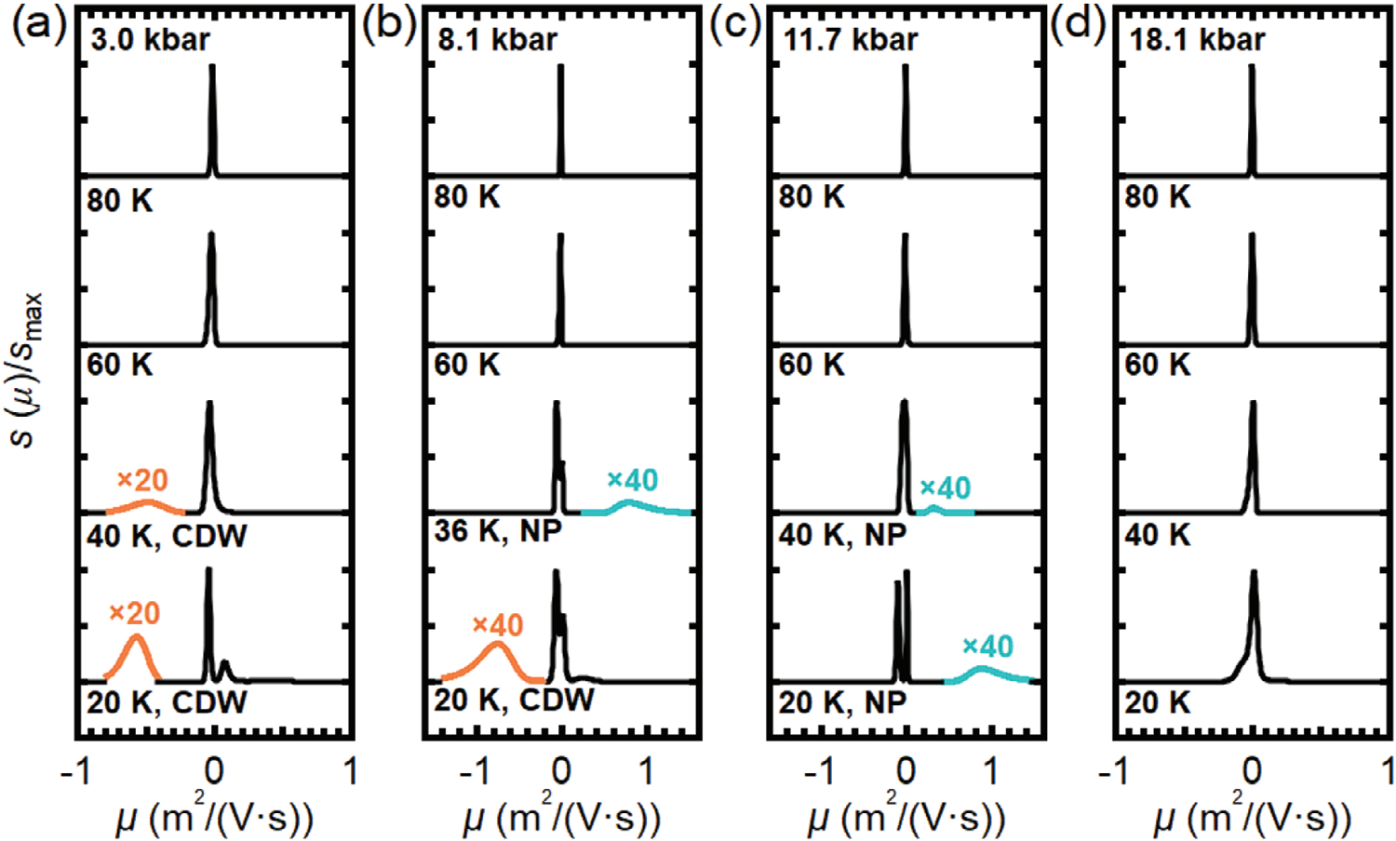
Mobility spectra of S2 at (a) 3.0 kbar, (b) 8.1 kbar, (c) 11.7 kbar and (d) 18.1 kbar. Each pressure includes mobility spectra at 80 K, 60 K, 40 K (except (b), where the spectrum at 36 K is shown), and 20 K with *s*(*μ*) renormalized as *s*(*μ*)/*s*_max_, where *s*_max_ is the maximum value of *s*(*μ*) in each spectrum. Orange and green curves denote elements related to the CDW and T*‐related phase, respectively, with their amplitudes magnified for clarity.

We further examine our transport data using the mobility spectrum analysis (MSA), which avoids any a priori assumptions about carrier types and numbers, and it provides detailed information on carrier mobilities.^[^ ^43^, ^44^ ^]^ The mobility spectra derived from the data on S2 are presented in **Figure** **3**, covering a wide range of temperatures at four representative pressures. Additionally, a complete set of mobility spectra is plotted on the semilog scale in the Supporting Information.^[^ ^38^ ^]^ For the pristine phase at 18.1 kbar, the mobility spectrum changes from having only a single peak close to *μ* = 0 at high temperatures, to displaying multiple peaks at low temperatures. This evolution is consistent with previous reports that the high‐temperature transport is dominated by one electron‐like band while multiband transport becomes active at low temperatures.^[^ ^14^, ^16^, ^45^ ^]^ Other spectra in the pristine phase are broadly consistent with the data at 18.1 kbar. For example, the spectrum at 80 K and 11.7 kbar also exhibits a single peak near *μ* = 0.

New structures emerge in the mobility spectra when T* and *T*_CDW_ start to play a role in transport. At 11.7 kbar, where T* is 43 K, a peak abruptly appears at around 0.3 m^2^/Vs when cooling to 40 K and further evolves to 0.9 m^2^/Vs at 20 K (Figure 3c). One possible interpretation is that a group of high‐mobility holes emerge in the T*‐related phase, and this set of holes has a significantly lower density, explaining the negligible effect introduced by T* on the electronic structure. Alternatively, the entrance into the T*‐related phase introduces a subtle effect to ρ_yx_(*B*), which manifests as a new peak in the mobility spectrum. To unambiguously settle this issue is difficult, because the new peak is very weak. If the positive peak were to manifest a group of holes, the Fermi surface should also undergo reconstruction upon entering the new phase. However, this contradicts the evidence from our quantum oscillation (as discussed later) and Hall resistivity results, which suggest no Fermi surface reconstruction at T*. Therefore, the positive peak is likely to have a different physical origin which requires further investigation. Nonetheless, this isolated peak structure provides a useful means to identify the T*‐related phase. At 8.1 kbar, at which both T* and *T*_CDW_ exist, new structures are observed in the mobility spectra. As shown in Figure 3b, at 80 and 60 K, a single‐peak structure appears near *μ* = 0 as expected, indicating the system stays in the pristine phase. Cooling to 36 K, a group of high‐mobility holes is found, signaling the stabilization of the T*‐related phase. Further cooling to 20 K, which is lower than *T*_CDW_, a new peak suddenly appears on the negative mobility side, centered at around −0.7m^2^/Vs. Phenomenologically, this new peak can be associated with the “S”‐shape feature found in the Hall signals in the CDW phase, because the “S”‐shape feature has a large, negative slope in the low field region, and the MSA routine interprets it as a group of high‐mobility electrons. However, since the “S”‐shape feature is recognized as the characteristic of the AHE in the AV_3_Sb_5_ family,^[^ ^14^, ^15^, ^16^, ^42^ ^]^ we adopt the mainstream interpretation that the new mobility peak is associated with the AHE.^[^ ^32^, ^46^ ^]^ Nevertheless, the high mobility peak on the negative side still serves as an indicator of the CDW phase. This indicator can also be found at 40 and 20 K at 3.0 kbar (Figure 3a), where *T*_CDW_ is 70 K. In conclusion, MSA provides diagnostic value for differentiating various phases in KV_3_Sb_5_, obtaining results agreeing with the *T*‐*p* phase diagram constructed using ρ(*T*).

Finally, we discuss the apparent relationship between the nonlinearity in ρ_yx_(*B*) at high field and the appearance of the high‐mobility peak on the positive mobility side, and whether the nonlinearity can be used as an indicator for the T*‐related phase. Our ρ_yx_(*B*) appears to deviate from linearity at the high‐field end below T*, see for example the dataset in the T*‐related phase at 11.7 kbar. Since the MSA gives a high‐mobility peak below T*, it might be tempting to link the two observations. However, one can reconstruct ρ_yx_(*B*) using the mobility spectrum. By intentionally excluding the high‐mobility peak, nonlinearity can still be observed in the reconstructed ρ_yx_(*B*), consistent with experimental data. Full technical details are provided in the Supporting Information.^[^ ^38^ ^]^ Thus, the high‐mobility peak on the positive side is not the cause of the nonlinearity in ρ_yx_(*B*).

### 2.4 Comparison between SdH Oscillations and DFT Calculation Results

To gain deeper insights into the new phase, we searched for clues from the perspective of electronic structures. We compare the electronic structure in the T*‐related phase with the electronic structures in other phases in KV_3_Sb_5_.

**Figure 4:**
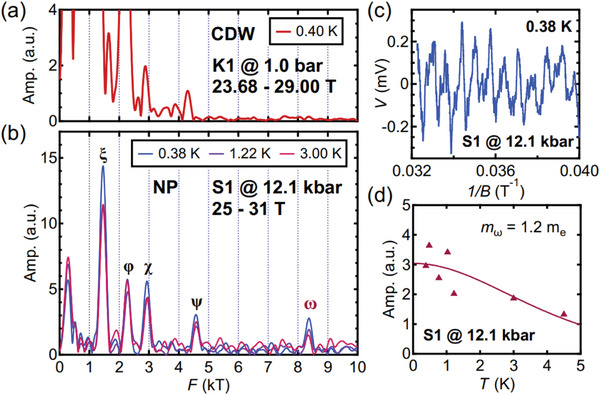
(a) FFT spectrum of the SdH oscillation data collected on K1 at 0.4 K at ambient pressure from 23.68 to 29.00 T. (b) FFT spectra of the SdH oscillation data collected on S1 at 12.1 kbar from 25 T to 31 T at three typical temperatures. Peaks are marked with ξ ∼ 1453 T, φ ∼ 2277 T, χ ∼ 2930 T, ψ ∼ 4577 T and ω ∼ 8372 T. The magnetic field is aligned along the *c*‐axis when conducting measurements in (a) and (b). (c) Oscillations of resistive voltage collected on S1 at 0.38 K with the background subtracted. (d) Temperature dependence of the amplitudes of ω derived from the FFT spectra collected on S1. The solid curve corresponds to Lifshitz–Kosevich analysis.

To begin with, we revealed the electronic structure of the CDW phase by performing high‐field Shubnikov‐de Haas (SdH) measurements on another thin flake sample (K1) up to 29 T and down to 0.4 K at ambient pressure, which undergoes a Fermi surface reconstruction at *T*_CDW_. The corresponding fast Fourier transform (FFT) analysis focusing on frequencies over 2000 T is shown in **Figure** **4**a, while the spectrum exhibiting all frequencies is presented in the Supporting Information.^[^ ^38^ ^]^ The result is broadly consistent with the published data in Ref. [39] except the appearance of frequencies at around 2900 T and around 4300 T, which are considered as second harmonics as explained later. Notably, no frequency larger than ∼4300 T is recorded. Next, we explored the electronic structure of the T*‐related phase by measuring the magnetoresistance up to 31 T in another pressure cell, in which we carefully increased the pressure of S1 to 12.1 kbar directly without going through any CDW phase transition. As described earlier, only the T*‐related anomaly was detected at 12.1 kbar in the normal state of S1, and the value of T* agrees with T*(*p*) of S2 (Figure 1c). The FFT spectra of the SdH data from 25 to 31 T are presented in Figure 4b, using the same horizontal scale as Figure 4a, which enables direct comparison between these two panels. In Figure 4c, we plot a representative oscillatory dataset (S1 at 12.1 kbar, 0.38 K) in the inverse field domain, the FFT of which is included in Figure 4b. In all high‐field measurements above, the magnetic field was applied along the *c*‐axis. Comparing S1 with K1, and S1 with the published data in Ref. [39], we make two remarks about the spectrum at 12.1 kbar: (i) the FFT spectrum of the present study is significantly simpler, and (ii) a large frequency of 8372 T (marked with ω), which is not revealed at ambient pressure, is observed. Finally, the corresponding quasi‐particle effective mass of ω is estimated by Lifshitz–Kosevich analysis, as shown in Figure 4d.

The SdH frequency at 8372 T is reminiscent of the large extremal orbit revealed recently by quantum oscillations in CsV_3_Sb_5_. In CsV_3_Sb_5_, a large SdH frequency (∼8200 T) has recently been detected in the high‐pressure “pristine” metallic state, i.e., when the CDW transition is tuned away.^[^ ^47^ ^]^ The CDW order introduces an in‐plane superlattice modulation resulting in a unit cell that is (2 × 2) times larger. Consequently, the Brillouin zone area in the *k*_*x*_‐*k*_*y*_ plane is reduced to approximately 4100 T for AV_3_Sb_5_. In both cases, the reconstructed Brillouin zone areas are too small to host an in‐plane extremal orbit corresponding to a frequency as large as 8 kT. Thus, the detection of such large frequencies can be regarded as an indicator for the absence of the CDW order. Hence, it is tempting to assign the observed 8372 T frequency in KV_3_Sb_5_ at 12.1 kbar to the large Fermi surface of the pristine metallic state, akin to CsV_3_Sb_5_, even though at 12.1 kbar the T*‐related phase is present.

**Figure 5:**
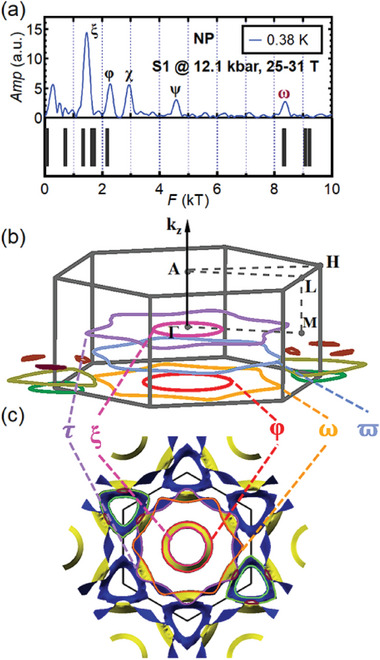
(a) Comparison between the SdH frequencies at 12.1 kbar and 0.38 K of S1 (upper panel) and the frequencies of Band 124 proposed by DFT calculations in the undistorted pristine phase (lower panel). The magnetic field is aligned along the *c*‐axis. b) Calculated extremal orbits parallel to the $k_{x}-k_{y}$ plane for Band 124. Only orbits with *k*_z_ ⩽ 0 are plotted for clarity. (c) Top views of the Fermi surfaces of Band 124 with the extremal orbits superimposed. The same color scheme is used for the extremal orbits in (b) and (c).

To investigate the fermiology of the pristine metallic state, we perform density functional theory calculations. The experimental data at 12.1 kbar and calculated frequencies are presented in **Figure** **5**a. In Figure 5b, the extremal orbits at various *k*_*z*_ are shown. We also show the top views of the Fermi surfaces within the first Brillouin zone in Figure 5c. Interestingly, the calculated results display a good agreement with the experimentally detected SdH frequencies at 12.1 kbar, despite the fact that the calculations do not assume any symmetry‐lowering order. For peaks labeled by ξ, φ, and ω, the agreement between the experimental results and calculations is particularly good (Figure 5a). The corresponding calculated extremal orbits can be seen in Figure 5b,c, with ω occupying nearly 52 % of the Brillouin zone area. The orbits labeled by τ and ϖ, which exceed 9000 T according to calculations, are not observed in our experiments. We hope that by enhancing the signal‐to‐noise ratio further, these frequencies can be detected in the future. No calculated values can be assigned to χ and ψ. Given that χ and ψ approximate twice the values of ξ and φ, they could be the second harmonics. Through the thermal damping factor of the Lifshitz–Kosevich theory, the quasiparticle effective masses (m*) can be calculated (see Figure S4, Supporting Information). Unfortunately, the relatively weak amplitudes prevent a conclusive verification of the harmonic relationship through the values of m*. Overall, the good agreement between the DFT calculation in the pristine phase and the experimental results in the T*‐related phase emphasizes that the Fermi surface is not reconstructed in the new phase.

## 3 Discussion

We now discuss the nature of the T*‐related phase based on existing experimental data. An evolution of the CDW phase from a commensurate long‐range order to an incommensurate order under pressure has been proposed by nuclear quadrupole resonance (NQR)^[^ ^25^ ^]^ in CsV_3_Sb_5_. It is conceivable that KV_3_Sb_5_ also enters an ICCDW order at T*, but T* has a different pressure dependence from the commensurate CDW order denoted by *T*_CDW_. Such a temperature‐pressure phase diagram has been constructed for 1T‐TaS_2_.^[^ ^48^, ^49^ ^]^ However, it remains difficult to explain why T* is not observed at ambient pressure, although it could be very close to *T*_CDW_ at low pressures such that it is hard to distinguish them. Importantly, the Fermi surface is expected to be reconstructed across the incommensurate CDW transition too – this has not been observed experimentally, based on our Hall data and quantum oscillations.

Another possibility arises from the spin degrees of freedom. Very recently, the coexistence of SDW phase, CDW phase and superconductivity has been observed in the Cr‐based kagome metal CsCr_3_Sb_5_ under pressure.^[^ ^50^ ^]^ The reported *T*‐*p* phase diagram is remarkably similar to the present case, thus it is reasonable to postulate an SDW transition at T* in KV_3_Sb_5_. However, at ambient pressure the metallic states preceding the superconductivity are different in CsCr_3_Sb_5_ and KV_3_Sb_5_. In CsCr_3_Sb_5_, an antiferromagnetic (AFM) state has been confirmed by nuclear magnetic resonance.^[^ ^50^, ^51^ ^]^ In KV_3_Sb_5_, however, evidence is accumulating that long‐range magnetic order does not exist.^[^ ^2^, ^17^, ^52^ ^]^ In particular, muon spin spectroscopy reported the absence of local moments in KV_3_Sb_5_ .^[^ ^53^ ^]^ Because of the multiband nature of KV_3_Sb_5_, an SDW order should reconstruct the complicated Fermi surface, which has not been observed in the Hall data and quantum oscillations. Thus, the SDW is also unlikely to be the T*‐related phase.

The need to preserve the pristine Fermi surface in the T*‐related phase imposes a strong constraint on the possible nature of the electronic state. A leading candidate is an electronic nematicity, which has been proposed in AV_3_Sb_5_ compounds.^[^ ^5^, ^30^, ^31^, ^35^, ^36^ ^]^ Nematicity breaks rotational symmetry but preserves translational symmetry. The fact that T* has not been observed in bulk KV_3_Sb_5_. ^[^ ^27^ ^]^ but appears in our thin flakes lends support to this scenario, because in the bulk sample multiple nematic domains can weaken signals from experimental probes that lack spatial resolution, due to the averaging effect. In our experiment, not only the thickness is significantly reduced but the separation between the voltage leads is also small (10 µm), leading to the possibility of probing only a dominant nematic domain in which the nematic order parameter is uniform. The relation between the sample size and the nematicity is explored earlier in CsV_3_Sb_5_ ,^[^ ^35^ ^]^ in which the two‐fold torque signals were detected only in small samples below ∼100 K.

In KV_3_Sb_5_, one can envisage a distortion of the Fermi surface from the perfect six‐fold symmetry, thereby breaking the rotational symmetry. While the shape of the Fermi surface is deformed in this manner, the cross‐sectional area can be preserved. Thus, the quantum oscillation frequency does not vary and certainly does not exhibit a discontinuity. Such a smooth evolution of the quantum oscillation frequency across a nematic phase transition has been observed. For instance, when FeSe is driven across the nematic phase transition by sulfur substitution, the largest extremal orbit δ evolves smoothly and changes from an ellipse (rotational symmetry broken) in the nematic phase to a circle (rotational symmetry restored) outside the nematic region.^[^ ^54^ ^]^ Such a scenario, proposed by DFT and confirmed by quantum oscillations for the case of FeSe,^[^ ^54^ ^]^   is consistent with our observation for KV_3_Sb_5_.

In conclusion, we discover a new phase in thin flakes of KV_3_Sb_5_ and trace its evolution through magnetotransport. The new phase emerges at low pressure and persists beyond the pressure at which the CDW phase is completely suppressed. The Hall measurements imply the absence of a severe Fermi surface reconstruction when cooling across the phase boundary. The comparison between quantum oscillation results and DFT calculations further confirms the preservation of the pristine metallic Fermi surface in the new phase. Based on our experimental evidence, we tentatively attribute the new phase to nematicity. Given that both the newly discovered phase and the CDW phase are in proximity to the superconducting phase, it is crucial to confirm the broken symmetry associated with the new phase to understand the dome‐shaped dependence of the superconducting transition temperature. It is also pressing to investigate the role of pressure in tuning the new phase, and explore the existence of this phase in sister compounds RbV_3_Sb_5_ and CsV_3_Sb_5_. The discovery of the new phase in KV_3_Sb_5_ provides a prime confirmation that the kagome lattice is a prolific platform for realizing multiple exotic phases.

## 4 Experimental Section

### Sample Preparation and Measurements

Single crystals of KV_3_Sb_5_ used in this work were synthesized using the self‐flux method as described in Ref. [39] Three KV_3_Sb_5_ thin flakes with similar thicknesses of around 200 nm, S1, S2 and K1 were cleaved from bulk single crystals from the same batch. While high‐pressure magnetotransport measurements of S1 and S2 were conducted with a device‐integrated diamond anvil cell (DIDAC) method,^[^ ^42^, ^55^, ^56^ ^]^ K1 was measured at ambient pressure. Both S1 and S2 were measured in a Physical Property Measurement System (PPMS) by Quantum Design, which provides temperature down to 2 K and magnetic field up to 14 T, while additional studies were conducted on S1 down to 0.38 K and up to 31 T at National High Magnetic Field Laboratory (NHMFL) in Tallahassee. Finally, K1 was studied down to 0.4 K and up to 29 T at Laboratoire National des Champs Magntiques Intenses (LNCMI) in Grenoble.

### Mobility Spectrum Analysis

The mobility spectrum analysis (MSA)^[^ ^43^, ^44^ ^]^ was conducted by combining both transverse magnetoresistivity and conventional Hall resistivity. The magnetotransport data were collected in S2 with the magnetic field applied along the *c* direction. From experiments, the resistivity tensor components ρ_*xx*_ and ρ_*yx*_ were obtained. They are then converted into conductivity tensor components σ_*xx*_ and σ_*xy*_. MSA takes σ_*xx*_ and σ_*xy*_, and outputs the mobility spectrum. The spectrum attributes the transport to the presence of carriers of different mobilities. The MSA method was successfully applied on FeSe and PtBi_2_. For FeSe, MSA enabled the detection of the high‐mobility electrons.^[^ ^57^, ^58^ ^]^ For PtBi_2_, MSA confirmed the presence of five bands in agreement with first principle calculation and ARPES measurement.^[^ ^59^ ^]^ There are multiple approaches to the MSA.^[^ ^60^ ^]^ In this paper, the maximum entropy approach was adopted introduced by Kiatgamolchai et al.^[^ ^43^ ^]^

### Density Functional Theory Calculations

The WIEN2k package,^[^ ^61^ ^]^ which employs density functional theory (DFT) with full‐electron full‐potential linearized augmented plane waves, was used in the Fermi surface calculation. The lattice constants of KV_3_Sb_5_ were adopted from Ref. [21] and no structural optimization was performed. Further details of the DFT calculation can be found in the Supporting Information S1.

## Conflict of Interest

The authors declare no conflict of interest.

## Supporting information

Supporting Information

## Data Availability

The data that support the findings of this study are available from the corresponding author upon reasonable request.
