# Supplementary material for: Discovery of a New Phase in Thin Flakes of KV3Sb5 under Pressure
Source: Adv Sci (Weinh). 2025 Feb 28;12(16):2415012. doi: 10.1002/advs.202415012 (PMC12021104; doi:10.1002/advs.202415012)
Supplement: Supplementary file 1 — Supporting Information [file ADVS-12-2415012-s001.pdf]

ADVANCED SCIENCE
Open Access

# Supporting Information

for *Adv. Sci.*, DOI 10.1002/advs.202415012

Discovery of a New Phase in Thin Flakes of $KV_3Sb_5$ under Pressure

*Zheyu Wang, Lingfei Wang, King Yau Yip, Ying Kit Tsui, Tsz Fung Poon, Wenyan Wang, Chun Wai Tsang, Shanmin Wang, David Graf, Alexandre Pourret, Gabriel Seyfarth, Georg Knebel, Kwing To Lai, Wing Chi Yu, Wei Zhang* and Swee K. Goh**

# Discovery of a New Phase in Thin Flakes of $KV_3Sb_5$ under Pressure

Zheyu Wang,[1] Lingfei Wang,[1] King Yau Yip,[1] Ying Kit Tsui,[1] Tsz Fung Poon,[1] Wenyan Wang,[1] Chun Wai Tsang,[1] Shanmin Wang,[2] David Graf,[3] Alexandre Pourret,[4] Gabriel Seyfarth,[5] Georg Knebel,[4] Kwing To Lai,[1] Wing Chi Yu,[6] Wei Zhang,[1,*] and Swee K. Goh[1,†]

[1] *Department of Physics, The Chinese University of Hong Kong, Shatin, Hong Kong, China*
[2] *Department of Physics, Southern University of Science and Technology, Shenzhen, Guangdong, China*
[3] *National High Magnetic Field Laboratory, Florida State University, Tallahassee, FL, USA*
[4] *Univ. Grenoble Alpes, CEA, Grenoble-INP, IRIG, Pheliqs, 38000 Grenoble, France*
[5] *Univ. Grenoble Alpes, INSA Toulouse, Univ. Toulouse Paul Sabatier, EMFL, CNRS, LNCMI, Grenoble 38042, France*
[6] *Department of Physics, City University of Hong Kong, Kowloon, Hong Kong, China*

(Dated: January 17, 2025)

## I. Further details of density functional theory calculations

The WIEN2k package [S1], which employs DFT with full-electron full-potential linearized augmented plane waves, was used in the Fermi surface calculation. The lattice constants of $KV_3Sb_5$ were adopted from Ref. [S2] and no structural optimization was performed. The generalized gradient approximation of Perdew, Burke and Ernzerhof for the exchange-correlation potential [S3], $R_{\mathrm{MT}}^{\mathrm{min}}K_{\mathrm{max}} = 7.5$, a $k$-point mesh of $28\times28\times16$ in the first Brillouin zone were utilized in the calculation. The muffin-tin radius was set to 2.47 a.u. for K and V atoms, and 2.5 a.u. for Sb atoms. The second variational procedure was employed to include spin-orbit couplings [S4]. Quantum oscillation frequencies were extracted from as-calculated band structures without any energy shift using the Supercell K-space Extremal Area Finder (SKEAF) [S5], where the averaging threshold for the fractional difference in frequencies and the orbital distances was set to 0.01 and 0.05 respectively.

In addition to the extremal orbits from Band 124, which are shown in Fig. 5(b) and (c), Band 125 also crosses the Fermi energy of the pristine electronic band structure in $KV_3Sb_5$. The Fermi surface sheets from Band 125 are shown in Fig. S1. The calculated frequencies of Band 125 are all less than 100 T, and frequencies with such small values cannot be easily resolved in our high-field Shubnikov-de Haas (SdH) oscillation experiments. Nevertheless, these small frequencies do not affect our discussion on whether the pristine Fermi surface is reconstructed – the resultant Fermi surface of Band 125 is included here for completeness.

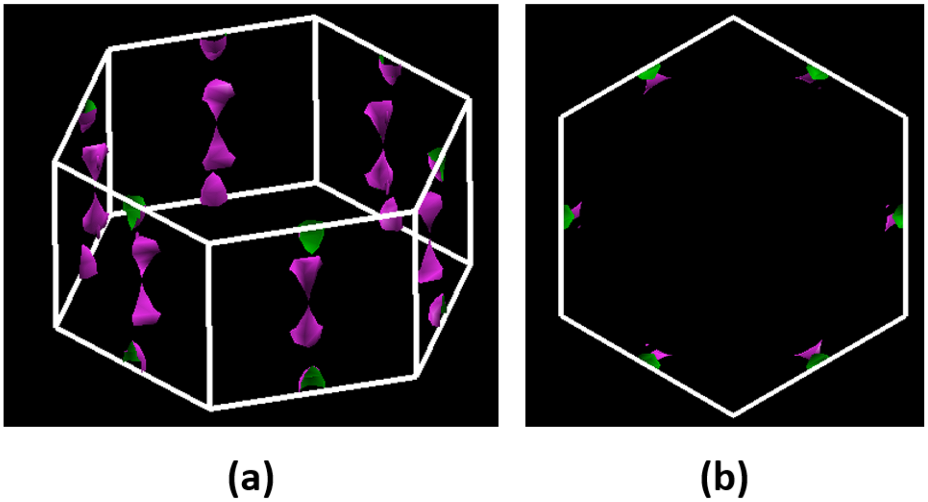


FIG. S1. The 3D plot (a) and top views (b) of the calculated orbits from Band 125.

Finally, we display the calculated band structure of the pristine phase of $KV_3Sb_5$ in Fig. S2, in which each level is doubly degenerated. Inspecting the band structure in the vicinity of the Fermi energy, it can be concluded that spin-orbit coupling has a negligible effect on the topography of the Fermi surface.

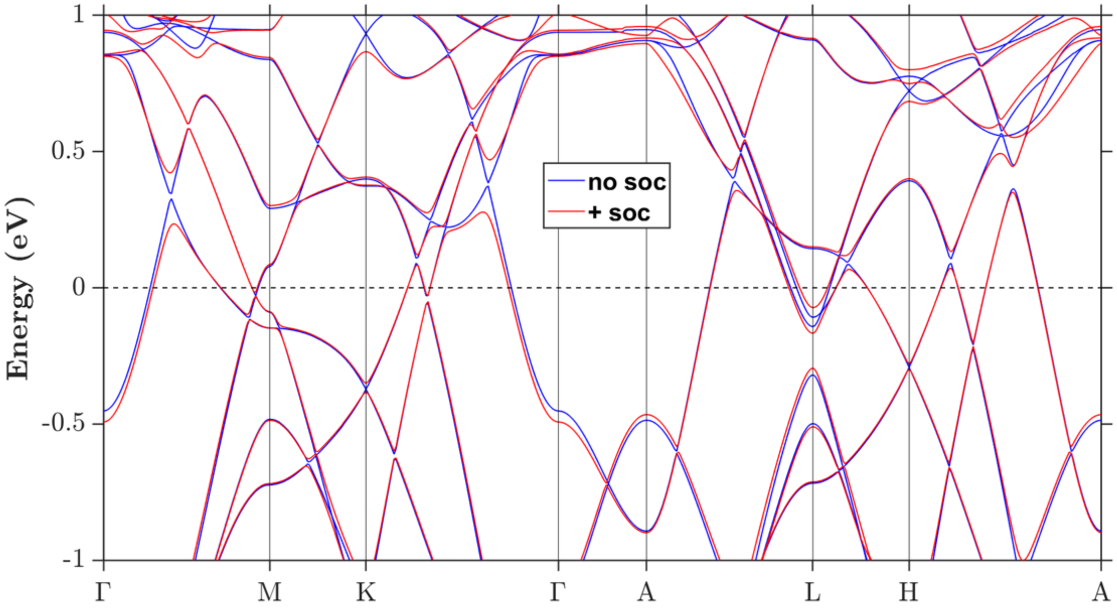


FIG. S2. The calculated band structure of the pristine phase in $KV_3Sb_5$. The red (blue) curve represents results with (without) considering spin-orbit couplings. Each level is doubly degenerated in the calculation.

## II. Additional Shubnikov-de Haas oscillation results

We present additional SdH oscillation results on S1 and K1 in this section. As mentioned in Section 2.4 in the main text, the fast Fourier transform (FFT) spectrum of the SdH data on K1 at 0.4 K and ambient pressure is displayed with the full vertical scale in Fig. S3. The group of low-frequency peaks is more visible with this scale, and the richer structure highlights the fact that $KV_3Sb_5$ at ambient pressure is in the charge density wave state.

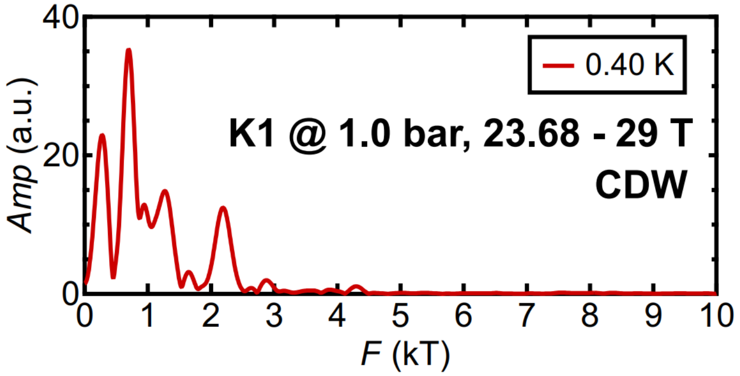


FIG. S3. FFT spectrum of the SdH oscillation data on K1 at ambient pressure and 0.4 K.

Then, we present the temperature dependence of the SdH oscillations at 12.1 kbar on S1, as shown in Fig. S4(a). Firstly, we carefully trace the temperature evolution of the SdH oscillation amplitudes at various temperatures ranging from 0.38 K to 8.0 K. The oscillation amplitudes are then extracted directly from Fig. S4(a) and plotted in Fig. S4(b) ($\xi$ and $\varphi$), Fig. S4(c) ($\chi$ and $\psi$) and Fig. S4(d) ($\omega$) as markers. Secondly, we obtain the effective masses according to the thermal damping factor $R_{\mathrm{T}}$ from the Lifshitz-Kosevich (L-K) theory [S6]:

$$R_T = \frac{14.693 m^* T/B}{\sinh\left(14.693 m^* T/B\right)}.$$

The corresponding fittings are presented as the solid curves in Figs. S4(b)–S4(d).

Next we attempt to verify the harmonic relationship between $\xi$ and $\chi$ and between $\chi$ and $\psi$ as proposed in the main text by comparing effective masses. Unfortunately, the relatively weak amplitudes prevent this effort. However, we can still have some insights by comparing the oscillation frequencies detected in the $T^*$-related phase in $KV_3Sb_5$ and the frequencies in the pristine phase of $CsV_3Sb_5$. As summarized in Table S1, except for $\xi$, each frequency found in $KV_3Sb_5$ has a corresponding frequency in $CsV_3Sb_5$ with a very similar value. Furthermore, the frequency of 4.59 kT ($2\delta$) has been proven to be the second harmonic of the frequency of 2.30 kT ($\delta$) in Ref. [S7]. The similar spectra detected in $KV_3Sb_5$ and $CsV_3Sb_5$ lend support to the harmonic relationship in $KV_3Sb_5$, as discussed in the main text. Finally, to emphasize the good agreement between the experiments and DFT calculations again, we summarize the SdH oscillation frequencies in $KV_3Sb_5$ and the DFT calculated results in the pristine phase in Table S2.

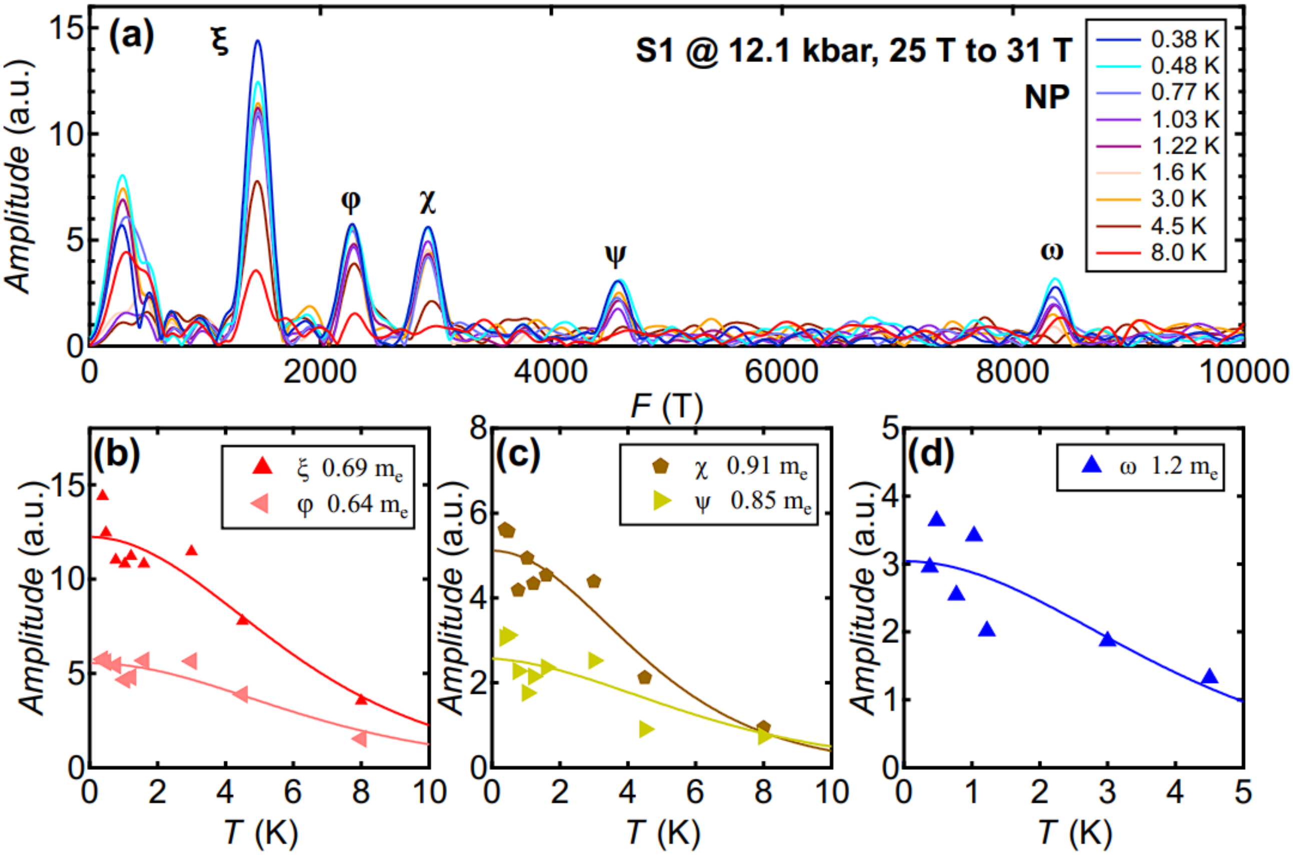


FIG. S4. (a) FFT spectra of the SdH oscillation data collected on S1 at various temperatures ranging from 0.38 K to 8.0 K at 12.1 kbar. The extracted oscillation amplitudes at various temperatures are presented as markers in (b) for $\xi$ and $\varphi$, (c) $\chi$ and $\psi$ and (d) $\omega$. The solid curves are fits based on the L-K theory.

| $KV_3Sb_5$ ($T^*$-related phase) | $CsV_3Sb_5$ (pristine phase) |
|---|---|
| $F$ (kT) | $F$ (kT) |
| | 0.15 |
| | 0.34 |
| 1.45 | 1.24 |
| 2.28 | 2.30 ($\delta$) |
| 2.93 | |
| 4.58 | 4.59 ($2\delta$) |
| 8.36 | 8.24 |

TABLE S1. Comparison between the experimental SdH oscillation frequencies detected in the $T^*$-related phase of $KV_3Sb_5$ and the frequencies detected in the high-pressure metallic pristine phase in $CsV_3Sb_5$. The data of $CsV_3Sb_5$ are collected at 24.5 kbar, from Ref. [S7].

| $KV_3Sb_5$ ($T^*$-related phase) | $KV_3Sb_5$ (DFT) |
|---|---|
| $F$ (kT) | $F$ (kT) |
| 1.45 | 1.32 |
| | 1.64 |
| | 1.71 |
| 2.28 | 2.17 |
| 8.36 | 8.33 |
| | 9.07 |
| | 9.22 |
| 2.93 | |
| 4.58 | |

TABLE S2. Comparison between the experimental SdH oscillation frequencies detected in the $T^*$-related phase of $KV_3Sb_5$ and the calculated frequencies in the pristine phase of $KV_3Sb_5$. Calculated frequencies below 1000 T are not included.

## III. Additional magnetotransport data collected on S1 and S2

We present additional magnetotransport data collected on S1 and S2 in this section. Firstly, the temperature dependence of resistance collected on S2 at all pressures are presented in absolute units in Fig. S5. This figure provides an alternative view of our data, complementing the presentation used in the main text in which renormalized resistivity data are used. Fig. S5(a) displays $R(T)$ curves ranging from 0 to 300 K, while Fig. S5(b) focuses on the data below 5 K. It is worth noting that, as clearly shown in Fig. S5(b), $T_c$ values are nearly the same immediately below (8.1 kbar) and above (11.7 kbar) the CDW phase boundary, which indicates a smooth evolution of $T_c$ when pressure changes across the CDW phase boundary. Furthermore, the superconducting transitions are sharp at 8.1 kbar and 11.7 kbar, as well as at higher pressures. These observations suggest that the $T^*$-related phase does not drastically affect the superconducting transition, compared with the superconducting transition within the regular CDW region.

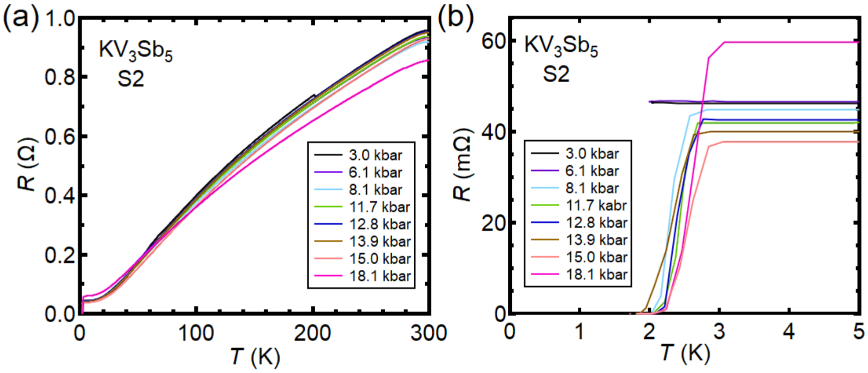


FIG. S5. Temperature dependence of resistance collected on S2 at all pressures, ranging from (a) 0 to 300 K and (b) 0 to 5 K.

Secondly, the renormalized resistivity of S1 and S2 collected respectively at two similar pressures, 12.1 kbar and 11.7 kbar, are shown in Fig. S6. It is worth noticing that the residual resistance and $T_c$ of both samples have similar values, as shown in the inset of Fig. S6(a). This can be understood considering that S1 and S2 have similar thicknesses and are cleaved from the same batch of bulk single crystals. Moreover, the geometries of the pre-patterned electrodes [S8, S9] used are the same. As a result, it is not surprising to find similar residual resistance and $T_c$ in Fig. S6(a). Besides, $T^*$ displayed in Fig. S6(b) have close values. This observation emphasizes that $T^*$, which has not been reported in previous studies, is an intrinsic pressure-induced energy scale that has been detected more than once by us in thin flakes of $KV_3Sb_5$.

The existence of both $T_{CDW}$ and $T^*$ observed in S2 at 8.1 kbar provides further evidence that $T^*$ is an energy scale independent from $T_{CDW}$. While the corresponding $d\rho/dT$ data are limited to 25 K in Fig. 1 in the main text, data covering a wider temperature range are shown in Fig. S6(c) for completeness, where $T_{CDW}$ and $T^*$ are benchmarked by the two minima in the curve. However, the measurement in Fig. S6(c) were conducted with a faster temperature sweeping rate, resulting in less accuracy in determing $T_{CDW}$ and $T^*$. Therefore, for consistency and accuracy, the phase transition temperatures from Fig. 1 with a slower sweeping rate are used for constructing the $T$-$p$ phase diagram.

Next, we present the Hall resistivity collected at 6.1 kbar and 12.8 kbar on S2 in Fig. S7(a) and (c). At 6.1 kbar only $T_{CDW}$ appears at 54 K in $d\rho/dT$. The 'S'-shape feature can be observed at 20 K and 40 K and can no longer be recognized at higher temperatures. This indicates the system stays in the CDW phase at 20 K and 40 K, and then the CDW order is gradually suppressed by temperature. The absolute value of the extracted Hall coefficient, $|R_H|$, decreases significantly between 40 K and 60 K, which is consistent with the Fermi surface reconstruction at $T_{CDW}$. At 12.8 kbar, only $T^*$ appears in $d\rho/dT$, and the Hall resistivity shown in Fig. S7(c) has very similar features as those shown in Fig. 2(c), which is collected at 11.7 kbar. As expected, the Hall coefficient in Fig. S7(d) changes smoothly when crossing $T^*$. To directly demonstrate the suppression of the anomalous Hall effect (AHE) by pressure, we extract the anomalous Hall resistivity from ordinary Hall signals at 20 K at various pressures. The method used for extraction is the same as the one used in Refs. [S10–S12]. As shown in Fig. S7(e), clear resistivity plateaus can be observed at 3.0 kbar, indicating the existence of AHE at 20 K. The resistivity plateaus are suppressed when the pressure increases to 6.1 kbar and 8.1 kbar and finally evolves to resistivity curves oscillating around zero, as shown in Fig. S7(f). The disappearance of the resistivity plateaus indicates the total suppression of CDW.

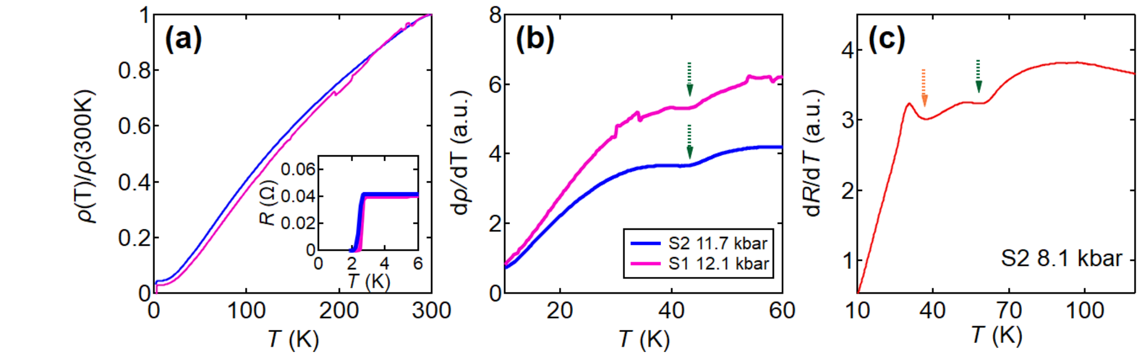


FIG. S6. Temperature dependence of (a) renormalized resistivity $\rho(T)/\rho(300\ \mathrm{K})$ and (b) $\mathrm{d}\rho/\mathrm{d}T$ collected on S1 at 12.1 kbar and on S2 at 11.7 kbar, respectively. The inset of (a) shows the sharp superconducting transition with close $T_\mathrm{c}$ in both samples. The green dashed arrows in (b) indicate $T^*$. The sudden jumps in $\rho(T)$ for S1 are experimental artefacts. However, the local minimum in $\mathrm{d}\rho/\mathrm{d}T$ at around 43 K for S1 can be unambiguously observed. All data are collected upon cooling. (c) $\mathrm{d}\rho/\mathrm{d}T$ collected on S2 at 8.1 kbar down to 10 K.

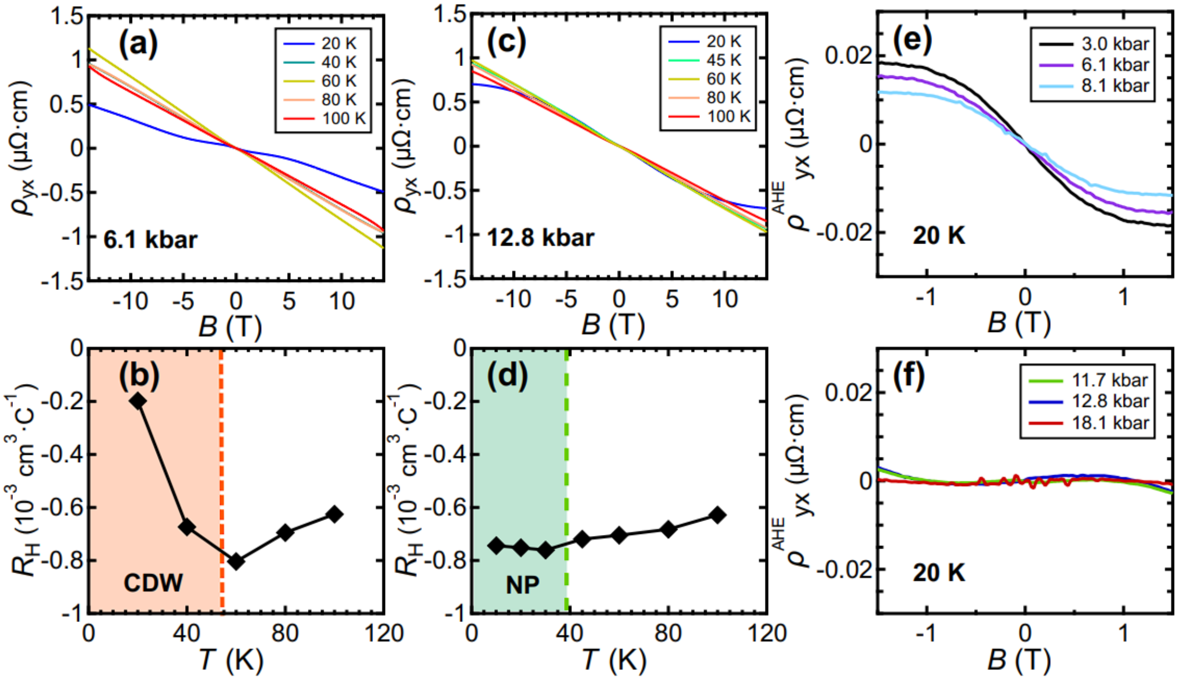


FIG. S7. (a), (c) Hall resistivity against the magnetic field at various temperatures collected on S2 at 6.1 kbar and 12.8 kbar, respectivety. (b), (d) Hall coefficients against the temperature extracted from the dataset in (a) and (c). The orange shaded area in (b) represents the CDW phase while the green shaded area in (d) represents the $T^*$-related phase. (e), (f) The anomalous Hall resistivity extracted from ordinary Hall resistivity at various pressures. The data in (e) and (f) are collected on S2 at 20 K.

## IV. Additional mobility spectrum analysis on S2

In addition to the MSA spectra described in Fig. 3 which covers only four pressures in the main text because of the space constraint, we present a complete set of mobility spectra on the semilog scale covering six pressures in Fig. S8.

We emphasize that the mobility spectra at 18.1 kbar serve as useful benchmark here, because both $T_{\rm CDW}$ and $T^*$ are tuned away at this pressure. Figure S8(u) shows the spectrum at 80 K, in which a sharp peak centered at a negative value near the origin is revealed, indicating a set of low-mobility electrons dominates the transport at 80 K. This is consistent with the linearity of $\rho_{\rm yx}$ with a negative slope. The single-peak feature persists and the width of the peak begins to increases when the temperature decreases to 60 K and 40 K. At 20 K, the peak becomes even wider and covers both positive and negative mobility values, indicating a new group of low-mobility holes begins to participate in the transport. This again explains the deviation from linearity in $\rho_{\rm yx}$, as presented in Fig. 2(d).

At 6.1 kbar, a group of 'high-mobility electrons' appear at 20 K and 40 K but they are absent at 60 K and 80 K, as shown in Figs. S8(e)-(h). Considering that the 'high-mobility electrons' work as the indicator of CDW, the MSA spectra suggests that the system stays in the CDW phase at 20 K and 40 K and enters the pristine phase at higher temperatures. Since at 6.1 kbar $T_{\rm CDW}$ is detected at 54 K by $d\rho/dT$, this observation agrees well with the discussion in the main text. At 12.8 kbar, the group of 'high-mobility holes' appear at 20 K and disappear at higher temperatures, as shown in Figs. S8(q)-(t). Since $T^*$=39.5 K at 12.8 kbar, the disappearance of the 'high-mobility holes' suggests a transition from the $T^*$-related phase to the pristine phase, which is also consistent with the discussion in the main text. The evolution of the mobility spectra structure at 12.8 kbar is similar to the case at 11.7 kbar, as analyzed in the main text. Overall, the additional data presented in this section support the analysis in the main text. The excellent agreement further strengthens the identification of both $T^*$ and $T_{\rm CDW}$ under pressure.

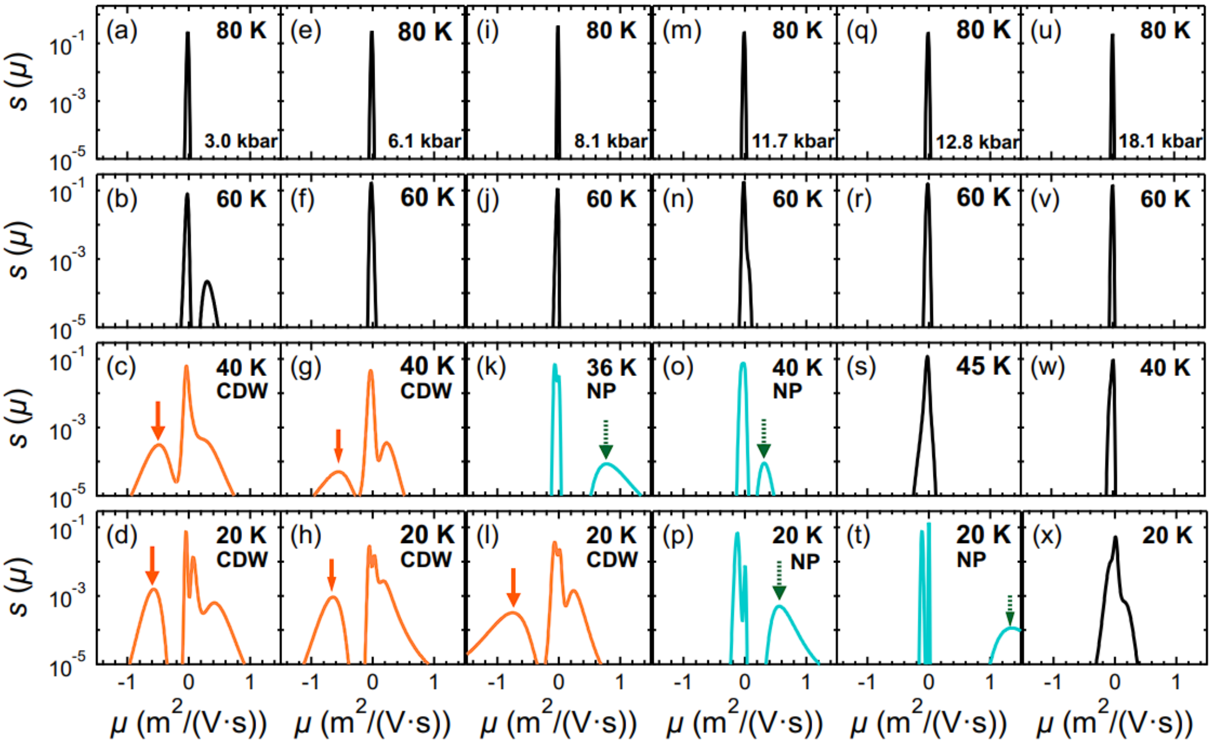


FIG. S8. MSA spectra of the transport data collected on S2 with $s(\mu)$ plotted in the log-scale at 3.0 kbar ((a)-(d)), 6.1 kbar ((e)-(h)), 8.1 kbar ((i)-(l)), 11.7 kbar ((m)-(p)), 12.8 kbar ((q)-(t)) and 18.1 kbar ((u)-(x)), respectively. The first row of panels represent MSA results at 80 K, while the second, third and the fourth row of panels respectively represent MSA spectrum at 60 K, 40 K (The only exception is (g), which is at 36 K) and 20 K. The black curves represent results in the pristine phase, while the orange and the green curves corresponds to results in the CDW phase and the $T^*$-related phase, respectively. The orange arrows indicate the feature related to CDW while the green arrows refer to the element representing the $T^*$-related phase.

## V. Exploring the origin of the nonlinearity in $\rho_{yx}$ at high field below $T^*$ by MSA

As mentioned in Section 2.3 in the main text, we have applied MSA to explore whether the deviation from linearity in the Hall resistivity at high field when $T^*$ emerges is induced by the high mobility peak on the positive mobility side, which also emerges in the $T^*$-related phase. This is achieved by reconstructing $\rho_{yx}(B)$ using information extracted from the mobility spectrum, while intentionally excluding the high mobility peak to examine whether the nonlinearity persists.

As a realistic example, we examine the Hall data at 20 K at 11.7 kbar (within the $T^*$-related phase), where deviation from linearity is the most pronounced among all datasets. In its corresponding mobility spectrum (Fig. 3(c) in the main text), three peaks are identified, and information of the carriers is extracted by fitting the spectrum with three Gaussian functions, which are labelled respectively from left to right by $g_1$, $g_2$ and $g_3$, as shown in Figure S9(a). Next, the $\rho_{yx}$ is simulated according to the following equations from Ref. [S13]:

$$\sigma_{xx} = \int_{-\infty}^{\infty} \frac{s(\mu)d\mu}{1+(\mu B)^2}$$

$$\sigma_{xy} = \int_{-\infty}^{\infty} \frac{\mu B s(\mu)d\mu}{1+(\mu B)^2}$$

$$\rho_{yx} = \frac{\sigma_{xy}}{\sigma_{xx}^2+\sigma_{xy}^2}$$

As shown in Fig. S9(b), the simulated Hall data that includes contributions from all three peaks (blue curve, $\rho_{yx}(g_1+g_2+g_3)$) is compared with the data that include contributions from only the two low-mobility peaks (red curve, $\rho_{yx}(g_1+g_2)$). Both the 'linearity' feature in the low-field region and the deviation from linearity are reproduced in both datasets. This simple exercise shows that the high mobility peak (and hence the emergence of the $T^*$-related phase) cannot be definitively attributed to the deviation from the linearity of $\rho_{yx}(B)$.

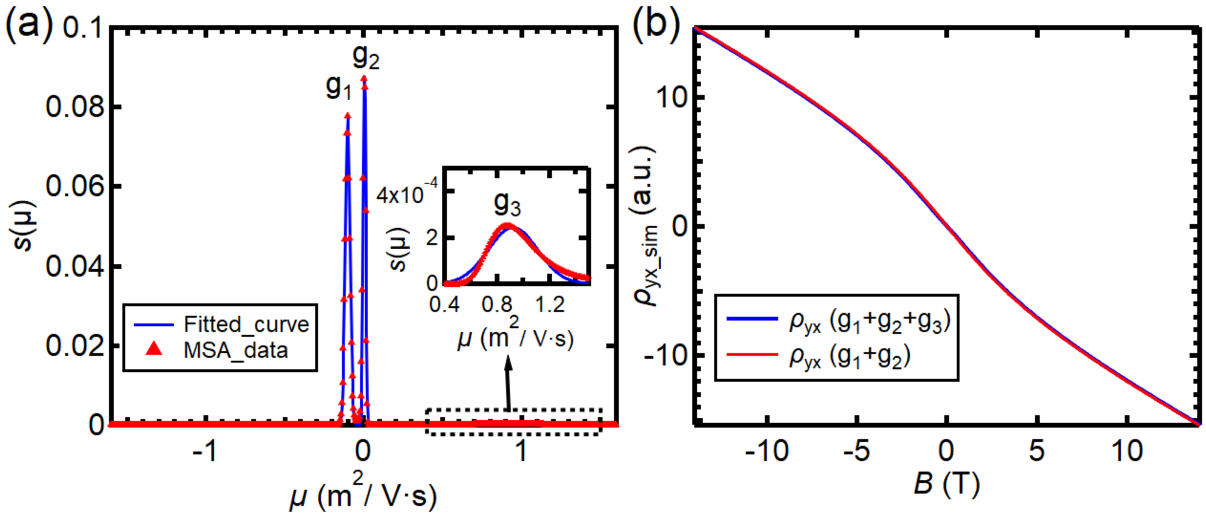


FIG. S9. (a) Extracting information of carriers from the mobility spectrum. The red markers represent the mobility spectrum at 11.7 kbar and 20 K while the blue curve represents the corresponding fitting results. The inset provides an expanded view of the high mobility peak. The Gaussian functions used for fitting are respectively labeled with $g_1$, $g_2$ and $g_3$ from left to right. (b) Simulated Hall resistivity corresponding to the mobility spectrum in (a). The blue curve includes the contributions from all three peaks, while the red curve excludes the contribution from the high mobility peak.

In fact, the deviation from linearity in $\rho_{yx}$ can be simply attributed to the multi-carrier transport by low-mobility electrons and holes. This multi-carrier transport nature has been confirmed in the pristine phase in $CsV_3Sb_5$ in Ref. [S12] and has been reported at ambient pressure and low temperatures in all three $AV_3Sb_5$ compounds in

Ref. [S14]. For these cases, no $T^*$ was reported. Hence, the departure from linearity is not specific to $T^*$-related phase, and does not provide a significant clue into the differences between the electronic structures of the $T^*$-related phase and the pristine phase.

* wzhang@phy.cuhk.edu.hk
† skgoh@cuhk.edu.hk

[S1] K. Schwarz and P. Blaha, "Solid state calculations using WIEN2k," Comput. Mater. Sci **28**, 259 (2003).
[S2] H. Tan, Y. Liu, Z. Wang, and B. Yan, "Charge density waves and electronic properties of superconducting kagome metals," Phys. Rev. Lett. **127**, 046401 (2021).
[S3] J. P. Perdew, K. Burke, and M. Ernzerhof, "Generalized gradient approximation made simple," Phys. Rev. Lett. **77**, 3865 (1996).
[S4] A. H. MacDonald, W. E. Picket, and D. D. Koelling, "A linearised relativistic augmented-plane-wave method utilising approximate pure spin basis functions," J. Phys. C. **13**, 2675 (1980).
[S5] P. Rourke and S. Julian, "Numerical extraction of De Haas–van Alphen frequencies from calculated band energies," Comput. Phys. Commun. **183**, 324 (2012).
[S6] D. Shoenberg, *Magnetic Oscillations in Metals.* (Cambridge Univ. Press, 1984).
[S7] W. Zhang, T. F. Poon, C. W. Tsang, W. Wang, X. Liu, J. Xie, S. T. Lam, S. Wang, K. T. Lai, A. Pourret, G. Seyfarth, G. Knebel, W. C. Yu, and S. K. Goh, "Large Fermi surface in pristine kagome metal $CsV_3Sb_5$ and enhanced quasiparticle effective masses," Proc. Natl. Acad. Sci. U.S.A **121**, e2322270121 (2024).
[S8] J. Xie, X. Liu, W. Zhang, S. M. Wong, X. Zhou, Y. Zhao, S. Wang, K. T. Lai, and S. K. Goh, "Fragile pressure-induced magnetism in FeSe superconductors with a thickness reduction," Nano Lett. **21**, 9310 (2021).
[S9] C.-h. Ku, X. Liu, J. Xie, W. Zhang, S. T. Lam, Y. Chen, X. Zhou, Y. Zhao, S. Wang, S. Yang, K. T. Lai, and S. K. Goh, "Patterned diamond anvils prepared via laser writing for electrical transport measurements of thin quantum materials under pressure," Rev. Sci. Instrum. **93**, 083912 (2022).
[S10] S.-Y. Yang, Y. Wang, B. R. Ortiz, D. Liu, J. Gayles, E. Derunova, R. Gonzalez-Hernandez, L. Šmejkal, Y. Chen, S. S. P. Parkin, S. D. Wilson, E. S. Toberer, T. McQueen, and M. N. Ali, "Giant, unconventional anomalous Hall effect in the metallic frustrated magnet candidate, $KV_3Sb_5$," Sci. Adv. **6**, eabb6003 (2020).
[S11] L. Wang, W. Zhang, Z. Wang, T. F. Poon, W. Wang, C. W. Tsang, J. Xie, X. Zhou, Y. Zhao, S. Wang, K. T. Lai, and S. K. Goh, "Anomalous Hall effect and two-dimensional Fermi surfaces in the charge-density-wave state of kagome metal $RbV_3Sb_5$," J. Phys. Mater. **6**, 02LT01 (2023).
[S12] F. H. Yu, T. Wu, Z. Y. Wang, B. Lei, W. Z. Zhuo, J. J. Ying, and X. H. Chen, "Concurrence of anomalous Hall effect and charge density wave in a superconducting topological kagome metal," Phys. Rev. B **104**, L041103 (2021).
[S13] W. A. Beck and J. R. Anderson, "Determination of electrical transport properties using a novel magnetic field-dependent Hall technique," J. Appl. Phys. **62**, 541 (1987).
[S14] X. Mi, W. Xia, L. Zhang, Y. Gan, K. Yang, A. Wang, Y. Chai, Y. Guo, X. Zhou, and M. He, "Multiband effects in thermoelectric and electrical transport properties of kagome superconductors $AV_3Sb_5$ (A = K, Rb, Cs)," New J. Phys. **24**, 093021 (2022).
